# Atrial Fibrillation and Heart Failure: Synergistic Effect on Functional Class and Quality of Life

**DOI:** 10.1002/clc.70113

**Published:** 2025-03-19

**Authors:** Sofía Zapata, Maria F. Colorado, Andrés Medina, Jaime A. Mejía, Sofia Betancur, Johanna M. Vanegas, James S. Díaz

**Affiliations:** ^1^ School of Health Sciences Universidad Pontificia Bolivariana Medellín Colombia; ^2^ Clínica Las Américas Auna Medellín Colombia

**Keywords:** atrial fibrillation, functional class, heart failure, left ventricular ejection fraction, prognosis, quality of life

## Abstract

**Background:**

Atrial fibrillation (AF) and heart failure (HF) are highly prevalent conditions associated with significant morbidity and symptom burden.

**Hypothesis:**

This study compared the evolution over time of functional class and quality of life (QoL) in patients with HF according to the presence of AF.

**Methods:**

A retrospective cohort study was conducted at an outpatient heart failure clinic in Colombia, between 2020 and 2022. Functional class (based on the New York Heart Association classification) and QoL (measured by the Minnesota Living with Heart Failure Questionnaire), were analyzed at baseline, 3 months, 6 months, and the last visit. The simultaneous impact of AF and left ventricular ejection fraction was analyzed using a generalized estimation equation model.

**Results:**

Among the 440 patients (median age 74 years, 56.6% men), 41.4% with AF, and 65.2% with reduced ejection fraction (HFrEF). Over time, functional class improved in both groups, with a more significant improvement in patients without AF. Patients with AF and HFrEF were more likely to remain in worse functional classes (OR: 2.77; 95% CI: 1.37–5.62). Similar trends were observed in QoL questionnaire, with sustained improvement after 3 months. However, AF negatively affected the physical dimension in patients with HFrEF, increasing the QoL questionnaire score by up to 4%.

**Conclusions:**

The presence of AF and reduced ejection fraction was associated with a lesser improvement in functional class and physical dimension of QoL questionnaire, emphasizing the importance of early detection and management of AF as part of comprehensive HF care.

## Graphical abstract

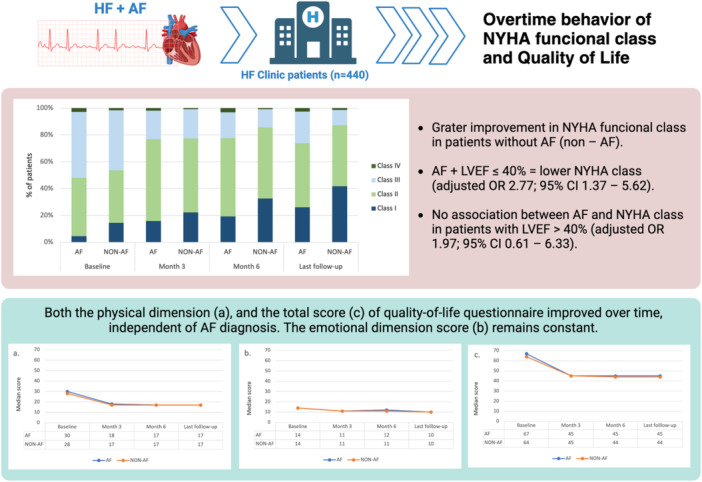

## 1 Introduction

Heart failure (HF) is a highly prevalent complex clinical syndrome. This condition is associated with a high rate of morbidity, mortality, and a significant burden of symptoms which deteriorate the quality of life (QoL) of patients. The characteristic symptoms of HF include dyspnea and physical fatigue that limits the ability to perform daily activities. Both symptoms result from structural or functional alterations in the heart associated with increased ventricular filling pressures [1]. Atrial fibrillation (AF) is the most common cardiac arrhythmia in clinical practice and is closely related to the risk of new HF or deteriorate of an existing chronic HF, worsens the prognosis of patients when both conditions coexistent [2, 3, 4, 5]. During the 30‐year follow‐up in the Framingham cohort, 57% of patients with new HF had concomitant AF, and 37% of those with new AF had HF [6]. Patients with HF and AF can experience a range of symptoms and diminished QoL [7, 8]. Despite the increase in the prevalence of both conditions and their interaction, few studies have evaluated the impact of AF on QoL and functional class in HF patients from low‐ and middle‐income countries. The objective of this study was to compare the evolution over time of functional class and QoL in patients with HF based on the presence of AF in a cohort of patients treated in a heart failure clinic in Colombia.

## 2 Methods

### 2.1 Type of Study and Population

This is an analytical observational study of a retrospective cohort of patients attended at the heart failure clinic of Clínica Las Américas Auna, a reference center for patients with cardiovascular diseases in Medellín (Colombia). In this outpatient program, a protocolized scheme for initiation and rapid titration of medications based on the recommendations of clinical practice guidelines is routinely offered. The protocol includes routine cardiac rehabilitation for all patients and evaluation by the electrophysiology service for patients with AF. There is availability of antiarrhythmics medications, ablation procedures, and cardiac devices when they are indicated. Functional class according to New York Heart Association (NYHA) classification and QoL according to the Minnesota Living with Heart Failure Questionnaire (MLHFQ) are measured in all patients at admission, 3, 6, and 12 months of follow‐up.

In this study, patients over 18 years old who were under follow‐up at the clinic during the years 2020 to 2022 were included. Based on the diagnosis of AF during the admission visit, two categories or groups of patients were created for comparison purposes. New cases of AF during the follow‐up period were not take on count for the analysis; however, according to previous own data, the frequency of new diagnoses of AF in this clinic is very low. Patients with less than 6 months of follow‐up and those lacking information on the functional class (NYHA classification) and QoL questionnaire (MLHFQ) at the initial were excluded. All patients who satisfied the eligibility criteria during the study period were consecutively included. The research protocol was approved by both research ethics committees of Clínica Las Américas Auna (approval No. 201, 2023) and Pontifical Bolivarian University (approval No. 21, 2022), only patients who authorized the use of information in medical records for clinical research, were included.

### 2.2 Variables and Follow‐Up

The analyzed outcomes were the change over time of the functional class according to the NYHA classification and QoL measured using the Minnesota Living with Heart Failure Questionnaire (MLHFQ) [1, 9]. The NYHA functional class was categorized as class I: no limitation for physical activity; class II: ordinary physical activity produces symptoms such as fatigue, dyspnea, or palpitations; class III: any physical activity produces symptoms; and class IV: symptoms even at rest. The MLHFQ has been previously validated for use in Colombia. It consists of 21 items that are grouped into a physical dimension (8 items), an emotional dimension (5 items), and other questions related to the signs and symptoms of the disease and the patient's functional status (8 items) [9]. Each item in the MLHFQ is scored from 0 to 5, where 0 means “no limitation” and 5 means “great limitation”. Both the NYHA classification and the MLHFQ were evaluated at four points of follow‐up: upon entering the heart failure clinic (baseline), month 3, month 6, and the last follow‐up visit. HF was defined according to universal definition [10]. The exposure variable was AF diagnosis at baseline, which should have been documented across a standard 12‐lead electrocardiogram, during 24‐h Holter monitoring or telemetry from a permanent cardiac device [3]. Additionally, the simultaneous effect of AF and systolic dysfunction (left ventricular ejection fraction [LVEF ≤ 40%]) was evaluated to determine a possible synergistic effect on the outcomes assessed. The other evaluated variables were age, sex, clinical history, presence of cardiac devices, and HF etiology.

### 2.3 Information Collection

Clinical history records and institutional databases were used as sources of information, and the unit of analysis was the patient. Additionally, an online form, which was reviewed by the group of researchers, was designed. This form has validation fields and closed questions to prevent errors during data entry. Data were retrospectively collected by researchers trained by cardiologists and nurses from the institutional program. The starting point (t0) was the date of the patient's first visit to the heart failure clinic, and changes in functional class and QoL were evaluated during follow‐up visits. Both the presence of AF and LVEF were evaluated at baseline.

### 2.4 Statistical Analysis

Qualitative variables were presented as absolute and relative frequencies and compared using the chi‐square test. For continuous quantitative variables, the assumption of normality was verified using the Shapiro‐Wilk test, and since it was not met, they were expressed with the median and interquartile range (IQR) and compared using the Mann‐Whitney U test. The association between AF and NYHA functional class was analyzed using a multilevel mixed‐effects ordered logistic regression to obtain the respective odds ratio (OR) with 95% confidence intervals (CI) and p‐values. The association between AF and changes over time in QoL was analyzed using a multilevel mixed‐effects generalized linear model with Gamma distribution and log link for estimating rates of change, along with 95% CI and p‐values. Each model was stratified according to the LVEF (≤ 40% or > 40%) and adjusted by time in the heart failure program, age, sex, cardiac devices and comorbidities (hypertension, diabetes, chronic obstructive pulmonary disease, chronic kidney disease, or cancer). A *p*‐value < 0.05 was considered statistically significant. The statistical analysis was conducted using STATA version 18.0.

## 3 Results

A total of 440 patients who met the eligibility criteria were included. Of them, 249 (56.6%) were men, with a median age of 74 years (IQR: 65–82). The clinical background was predominantly hypertension (62.5%), diabetes (36.1%) and chronic kidney disease (defined as a creatinine clearance <20 mL/min/1.73 m²–28.2%). The main etiology of HF was ischemic (64.8%), and most patients presented with systolic dysfunction (LVEF ≤ 40%–65.2%) (Table 1).

**Table 1:** Baseline characteristics of patients with heart failure according to the presence of atrial fibrillation.

Characteristic	Total (*n* = 440)	Atrial fibrillation		*p value*
	No (%)	Si (*n* = 182)	No (*n* = 258)	
**Sex**				0.011
Male	249 (56.6)	90 (49.5)	159 (61.6)	
Female	191 (43.4)	92 (50.5)	99 (38.3)	
**Age in years**				< 0.001
Median (IQR)	74 (65–82)	78 (72–85)	70 (63–79)	
**Marital status**				0.008
Single	98 (22.3)	34 (18.7)	64 (24.8)	
Common‐law marriage	36 (8.2)	11 (6.01)	25 (9.7)	
Marriage	196 (44.5)	77 (42.3)	119 (46.1)	
Widowed	110 (25.0)	60 (33.0)	50 (19.4)	
**Occupation**				0.002
Housewife	123 (27.9)	65 (35.7)	58 (22.5)	
Unemployed	66 (15.0)	20 (11.0)	46 (17.8)	
Employee	76 (17.3)	22 (12.1)	54 (20.9)	
Retiree	175 (39.8)	75 (41.2)	100 (38.8)	
**Smoking status**				0.280
Active	19 (4.3)	7 (3.8)	12 84.6)	
Previous	247 (56.1)	95 (52.2)	152 (58.9)	
Never	174 (39.5)	80 (44.0)	94 (36.4)	
**Clinical history**				
Arterial hypertension	275 (62.5)	113 (62.1)	162 (62.8)	0.881
Diabetes mellitus	159 (36.1)	67 (36.8)	92 (35.7)	0.804
Chronic obstructive pulmonary disease	75 (17.0)	36 (19.8)	39 (15.1)	0.200
Chronic kidney disease (CKD – CrCl < 20 mL/min/1.73 m²)	124 (28.2)	55 (30.2)	69 (26.7)	0.425
Solid cancer or hematological neoplasm	44 (10.0)	19 (10.4)	25 (9.7)	0.796
Obesity (BMI > 35 kg/m^2^)	85 (19.3)	31 (17.0)	54 (20.9)	0.308
**Cardiac devices**				0.002
Pacemaker	39 (8.9)	27 (14.8)	12 (4.6)	
CRT	22 (5.0)	12 (6.6)	10 83.9)	
ICD	46 (10.4)	15 (8.2)	31 (12.0)	
CRT – D	13 (2.9)	5 (2.7)	8 (3.1)	
None of the above	320 (72.7)	123 (67.6)	197 (76.4)	
**Heart failure etiology**				
Isquemic	285 (64.8)	48 (26.4)	107 (41.5)	0.001
Hypertensive	65 (14.8)	27 (14.8)	38 (14.7)	0.975
Arrhythmia – induce cardiomyopathy	30 (6.8)	26 (14.3)	4 (1.5)	< 0.001
Valvular cardiomyopathy	42 (9.5)	26 (14.3)	16 (6.2)	0.004
Idiopathic cardiomyopathy	42 (9.5)	18 (9.9)	24 (9.3)	0.836
Other etiologies	75 (17.0)	26 (14.3)	49 (19.0)	0.196
**Left ventricular ejection fraction (LVEF)**				< 0.001
Median (IQR)	35 (27–50)	40 (30–55)	34 (25–45)	
**Heart failure classification**				0.021
HFrEF (LVEF ≤ 40%)	287 (65.2)	105 (57.7)	182 (70.5)	
HFmrEF (LVEF 41%–49%)	38 (8.6)	19 (10.4)	19 (7.4)	
HFpEF (LVEF ≥ 50%)	115 (26.1)	58 (31.9)	57 (22.1)	

Abbreviations: BMI, Body mass index; CrCl, creatinine clearance; CRT, cardiac resynchronization therapy; CRT – D, Cardiac resynchronization therapy with a defibrillator; EF, ejection fraction; HF, heart failure; HFmrEF, heart failure with middle reduce ejection fraction; HFpEF, heart failure with preserved reduce ejection fraction; HFrEF, heart failure with reduce ejection fraction; ICD, Implantable cardioverter defibrillator; IQR, Interquartile range; LVEF, Left ventricular ejection fraction.

### 3.1 Atrial Fibrillation

The overall prevalence of AF was 41.4% (*n* = 182). When comparing the clinical characteristics according to the presence of AF, a higher proportion of women was found in the AF group (50.5% vs 38.3%; *p* = 0.011), as well as arrhythmia‐induced cardiomyopathy (14.3% vs 1.5%, *p* < 0.001) and valvular cardiomyopathy (14.3% vs 6.2%; *p* = 0.004). The median age (78 vs 70; *p* < 0.001) and median LVEF (40% vs 34%; *p* < 0.001) were also higher in patients with AF than in those without AF (Table 1).

### 3.2 NYHA Functional Class and Atrial Fibrillation

**Figure 1:**
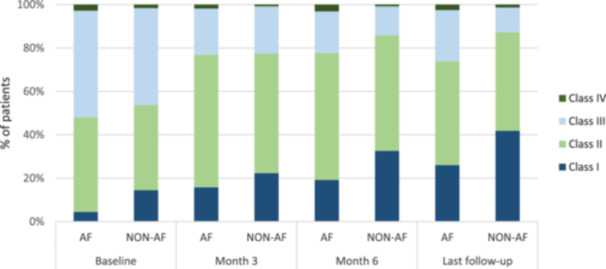
NYHA functional class according to the presence of atrial fibrillation during follow‐up. Baseline (*n* = 434), month 3 (*n* = 402), month 6 (*n* = 400), and last visit (*n* = 388).

In general, there was a trend toward improvement in the NYHA functional class during follow‐up, with a higher proportion of patients in class I and II after 6 months of follow‐up. However, comparing patients according to AF status, it was observed a greater proportion of patients without AF (non – AF) in NYHA I functional class at baseline and at the last follow‐up. At baseline, 4.5% of patients with AF versus 14.5% of non – AF patients were in class I; while for the last follow‐up visit, 26.1% of AF patients versus 41.8% of non – AF patients were in class I (Figure 1). The analyses of the simultaneous effect of AF and systolic function (left ventricular ejection fraction) on functional class showed that patients with AF and LVEF ≤ 40% tended to belong to poor functional classes (adjusted odds ratio: 2.77; 95% CI: 1.37–5.62; *p* = 0.005) while no association between AF and NYHA class was observed in patients LVEF > 40% (adjusted odds ratio: 1.97; 95% CI: 0.61–6.33; *p* = 0.253), Table 2.

**Table 2:** Association between atrial fibrillation and NYHA functional class at the last follow‐up visit, stratified by LVEF.

NYHA functional class	LVEF ≤ 40%		LVEF > 40%	
	OR (95% CI)	*p* value	OR (95% CI)	*p* value
Crude	2.37 (1.34–4.19)	0.003	1.87 (0.66–5.26)	0.235
Time‐adjusted	2.93 (1.44–6.00)	0.003	2.10 (0.66–6.68)	0.207
Multivariate*	2.77 (1.37–5.62)	0.005	1.97 (0.61–6.33)	0.253

* Adjusted by: Time, age, sex, cardiac devices and comorbidities (hypertension, diabetes, chronic obstructive pulmonary disease, chronic kidney disease, or cancer).

Abbreviations: LVEF, Left ventricular ejection fraction.

### 3.3 Atrial Fibrillation and QoL

**Figure 2:**
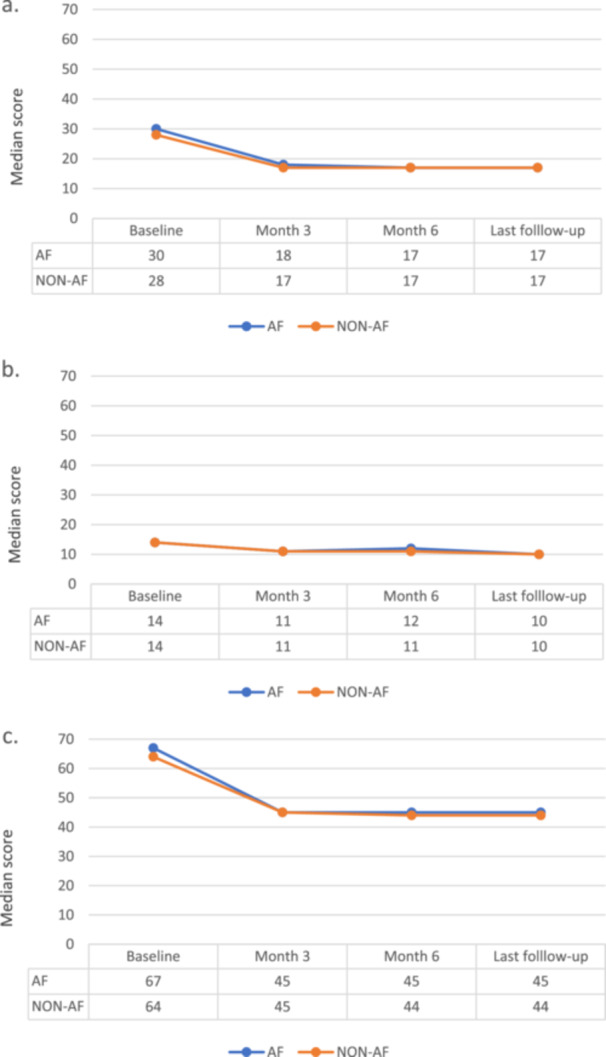
Quality of life according to the presence of atrial fibrillation. a: Physical dimension; b: Emotional dimension; c: Total Score. (Baseline *n* = 427, Month 3 *n* = 314, Month 6 *n* = 293, last visit = 259).

The behaviors over time for each dimension of QoL were similar independent of AF diagnosis. For both the total score and the physical dimension score of QoL questionnaire, an improvement was observed between baseline and the 3‐month follow‐up, with a stable trend at 6 months and in the last follow‐up visit (Figure 2). The analyses of the simultaneous effect of AF and systolic function (left ventricular ejection fraction) on QoL found a slight impact on the physical dimension, such that having both AF and LVEF ≤ 40% increased the score by up to 4% (adjusted rate of change: 1.04; 95% CI: 1.00–1.08; *p* = 0.036). This trend was not observed for the emotional dimension or for the total scale score (Table 3).

**Table 3:** Association between atrial fibrillation and quality of life, stratified by left ventricular ejection fraction.

MLHFQ score	LVEF ≤ 40%		LVEF > 40%	
	Rate of change (95% CI)	*p value*	Rate of change (95% CI)	*p value*
Physical dimension				
Crude	1.05 (1.00–1.09)	0.029	1.02 (0.96–1.08)	0.497
Time‐adjusted	1.04 (1.00–1.08)	0.058	1.02 (0.97–1.07)	0.440
Multivariate*	1.04 (1.00–1.08)	0.036	1.01 (0.97–1.06)	0.533
Emotional dimension				
Crude	1.02 (0.98–1.06)	0.292	1.00 (0.94–1.06)	0.909
Time‐adjusted	1.02 (0.98–1.06)	0.369	1.00 (0.94–1.06)	0.954
Multivariate*	1.02 (0.99–1.06)	0.200	1.00 (0.94–1.06)	0.998
Total score				
Crude	1.03 (0.99–1.06)	0.091	1.00 (0.96–1.04)	0.975
Time‐adjusted	1.02 (0.99–1.05)	0.162	1.00 (0.96–1.04)	0.946
Multivariate*	1.02 (0.99–1.06)	0.099	1.00 (0.96–1.04)	0.959

* Adjusted by: Time, age, sex, cardiac devices and comorbidities (hypertension, diabetes, chronic obstructive pulmonary disease, chronic kidney disease, or cancer)

Abbreviations: LVEF, Left ventricular ejection fraction.

## 4 Discussion

The central aim of this study was to analyze the temporal behavior of functional class and QoL in patients with HF, according to the AF diagnosis and systolic function (LVEF). Evaluated patient–related effects of symptoms from AF in patients with HF over time is recommended by clinical practice guidelines and improvements in QoL and functional status should play a key role in assessing and reassessing treatment decisions. The presence of AF intensifies symptoms of HF, such as dyspnea and fatigue, leading to physical limitation and deterioration of the functional class [3, 11]. Cardiac–specific AF symptoms, such as palpitations, are less common than nonspecific symptoms, such as fatigue; but both of them significantly impair the QoL [12, 13, 14].

In this single reference center study, the prevalence of AF was > 40%. Another Colombian registry reported a prevalence of AF of 22.28% in patients with HF [15]. In this cohort, we observed improvement in the NYHA functional class in all HF patients, regardless of the presence of AF. However, greater functional class improvement was observed in patients without AF compared with those with AF. On the other hand, it has been shown that the prognosis of patients with HF and AF may be affected by LVEF, with the rate of death highest with the combination of AF and HF with reduced ejection fraction (HFrEF–LVEF ≤ 40%), as compared with AF and HF with preserved EF (HFpEF–LVEF ≥ 50%) [16, 17]. Other studies have also found that the coexistence of AF and HFrEF increases the risk of symptoms and potential complications, which can lead to worse clinical outcomes [18, 19].

Other demographic characteristics involved in the clinical course of both HF and AF are age and sex. In this study of patients with HF, AF was predominantly observed in female patients, and the median age was > 70 years. This finding is important because although women are often underrepresented in clinical trials of AF, the available literature suggests that women with AF are more symptomatic and have poorer QoL [20, 21]. In addition, patients with AF report a higher burden of anxiety and severity of depression than the general population [22, 23] with the higher prevalence of these symptoms in women with HF [24].

The emotional and physical dimensions of the QoL questionnaire in HF patients tended to improve over time, regardless of the presence of AF. We observed that the physical dimension experienced greater improvement over time, with higher scores at baseline and a sustained improvement after 3 months of follow‐up, demonstrating the positive impact of comprehensive treatment in the heart failure clinic. Other studies have shown that a dedicated program for patients with HF resulted in significant improvements in physical function and QoL, indicating that it is possible to achieve substantial enhancements in patients' quality of life, particularly in the physical dimension, by implementing appropriate and continuous dedicated programs [25]. By including systolic function in this analysis, patients with HFrEF and AF showed greater impairment in the physical dimension, a result similar to a study that demonstrated that the physical component fundamentally determines the decrease in QoL of these patients [11].

Finally, concerning chronic diseases, such as hypertension, diabetes, and chronic kidney disease, these clinical backgrounds frequently coexisted in patients with HF and AF, contributing to its increase [17]. A broad array of comorbidities is associated with HF and AF recurrence and progression; driving comorbidities is also central to the success of other aspects of care. In our study, the most common condition in the population was hypertension, followed by diabetes and chronic kidney disease.

The results obtained from this study provide an important perspective on impact of AF in functional class and QoL in a cohort of patients with HF, with a close follow‐up to observe changes and trends over time. The study limitations include the recruitment of HF patients from a single reference center, which may impact the external validity of the findings. Additionally, other cointerventions offered at the clinic may account for the observed improvement in QoL over time. However, the results of this study highlight the importance of comprehensive management of AF improve the functional status in patients with HF.

## 5 Conclusion

The results of this research reinforce the impact of AF on the functional status in patients with HF. The simultaneous presence of AF and reduced ejection fraction was associated with a lesser improvement in functional class and reduced quality of life in its physical dimension, highlighting the importance of identifying and addressing early the AF management as part of comprehensive management of patients with HF.

## Ethics Statement

This study was performed in line with the principles of the Declaration of Helsinki. Only patients who had authorized the use of their clinical records for research purposes were included. Approval was granted by the institutional review boards (Health Research Ethics Committee of the Pontifical Bolivarian University with approval No. 21‐2022 and Clínica Las Américas Auna with approval No. 201‐2023).

## Consent

The authors confirm that patient consent is not applicable to this research, since it is a retrospective study that used secondary sources.

## Conflicts of Interest

The authors declare no conflicts of interest.

## Data Availability

Data available on request from the authors.
